# Hepatitis C Virus and Hepatocellular Carcinoma: When the Host Loses Its Grip

**DOI:** 10.3390/ijms21093057

**Published:** 2020-04-26

**Authors:** Kaku Goto, Armando Andres Roca Suarez, Florian Wrensch, Thomas F. Baumert, Joachim Lupberger

**Affiliations:** 1 Université de Strasbourg, F-67000 Strasbourg, France; 2 Institut National de la Santé et de la Recherche Médicale, U1110, Institut de Recherche sur les Maladies Virales et Hépatiques, Université de Strasbourg (IVH), F-67000 Strasbourg, France; 3 Pôle Hépato-digestif, Institut Hopitalo-Universitaire, F-67000 Strasbourg, France; 4 Institut Universitaire de France, F-75231 Paris, France

**Keywords:** HCV, HCC, epigenetics, signaling, tumor immunity, clinical impact

## Abstract

Chronic infection with hepatitis C virus (HCV) is a major cause of hepatocellular carcinoma (HCC). Novel treatments with direct-acting antivirals achieve high rates of sustained virologic response; however, the HCC risk remains elevated in cured patients, especially those with advanced liver disease. Long-term HCV infection causes a persistent and accumulating damage of the liver due to a combination of direct and indirect pro-oncogenic mechanisms. This review describes the processes involved in virus-induced disease progression by viral proteins, derailed signaling, immunity, and persistent epigenetic deregulation, which may be instrumental to develop urgently needed prognostic biomarkers and as targets for novel chemopreventive therapies.

## 1. Introduction

Globally, liver cancer is the sixth most commonly diagnosed cancer type and the fourth leading cause of cancer mortality [1,2]. With 70–80% of cases, hepatocellular carcinoma (HCC) is the most frequent liver cancer [1] and chronic infection with hepatitis C virus (HCV) has been recognized as a major cause of HCC [3]. In recent years, the direct-acting antiviral agents (DAAs) revolutionized the standard therapy, achieving high rates of sustained virologic response (SVR), which is associated with a largely reduced risk of mortality and HCC [4,5]. However, despite their efficacy, the novel therapies cannot fully eradicate liver cancer risk, especially in HCV-cured patients with advanced liver disease [6], suggesting an accumulation of irreversible damages to the liver during long-term HCV infection. Cirrhosis is an important factor in HCC development since the majority of HCV-associated HCCs occur in cirrhotic livers. Moreover, patients with established cirrhosis have a persistently elevated risk of HCC, even many years after SVR [4,7]. An association of DAA regimens with HCC development and recurrence was initially discussed but has not been confirmed by additional studies and meta-analyses [2,4,8,9]. Many studies suggest that the accumulation of liver damage during chronic HCV infection is caused by a complex interaction of direct and indirect mechanisms, which forces the liver towards a tilting point of no return in terms of HCC development. This review aims to summarize pro-oncogenic events induced by viral proteins, deranged host signaling, inflammation and immunity. Moreover, it highlights the role of epigenetic dysregulation by HCV and fibrosis in the pathogenic memory post infection, pointing towards novel and urgently needed biomarkers and chemopreventive concepts to identify and help patients at considerable HCC risk after cure.

## 2. HCV Life Cycle

HCV is an enveloped positive single-stranded RNA virus of the *Flaviviridae* family, which was discovered in 1989 as a cause of non-A, non-B hepatitis [10]. Viral particles expose heterodimers of the two viral glycoproteins E1 and E2, which are the main targets for neutralizing antibodies. Moreover, viral particles are associated with lipids and lipoproteins forming lipoviral particles of low and very-low buoyant density, which contributes to viral entry and a shielding from neutralizing antibodies [11]. HCV infects predominantly hepatocytes, but additional reservoirs in peripheral blood mononuclear cells (PBMC), including dendritic cells, B cells, and T cells have been suggested [12,13,14,15]. HCV entry in hepatocytes requires an unusually large number of host factors for binding, post-binding, internalization and fusion with endosomal membranes (reviewed in [16]), also engaging host signaling pathways involved in cell proliferation and survival. Translation of the released viral genome at the endoplasmic reticulum is initiated by a viral internal ribosomal entry site (IRES), which requires the microRNA (miRNA) miR-122 for its stabilization [17,18]. The viral polyprotein is then processed by host proteases and the viral proteases NS2/NS3 and NS3/4A to mainly ten viral proteins comprising structural proteins (core, E1, E2) and nonstructural (NS) proteins (p7, NS2, NS3, NS4A, NS4B, NS5A, NS5B) [19]. Viral proteins and the HCV-recruited host lipid kinase phosphatidylinositol 4-kinase III induce a deformation of the endoplasmic reticulum membranes to form the “membranous web”-termed replication complex [20]. The replication complex accumulates lipid droplets and lipoproteins, which are essential for virus assembly [19]. HCV lipoviral particles are released via the Golgi compartment in a non-lytic manner to the extracellular space [21] or are transmitted to the neighboring hepatocytes in a cell-free manner [16].

## 3. Pro-Oncogenic Impact of Viral Proteins

HCV does not code for classical viral oncogenes like v-src from Rous sarcoma virus [22] or E6/E7 from human papilloma virus [23]. However, some HCV proteins manipulate host pathways to favor tumor development by promoting cell proliferation and survival. Thus, HCV proteins contribute to a pro-oncogenic environment during chronic HCV infection [24]. Most of the evidence from cell culture and transgenic animal models supports the notion that mainly the HCV proteins core and the NS proteins 3 and 5A may have an active role in the development and progression of HCV-associated liver disease and HCC.

### 3.1. Core Protein

HCV core is an RNA-binding protein that, in combination with the viral genome, constitutes the nucleocapsid [25]. Core has been implicated in the development of several hepatic complications. In transgenic animal models, core expression alone is sufficient to induce hepatic steatosis [26], insulin resistance [27] and HCC [28]. It was suggested that core expression increases the production of reactive oxygen species (ROS), which results in an impaired mitochondrial β-oxidation [29]. These data indicate that HCV core promotes hepatocyte proliferation, which is emphasized by an accelerated liver regeneration following partial hepatectomy in core-transgenic mice [30]. This is further supported by the association of distinct core mutations in HCV genotype 1 with an elevated HCC risk in patients [31]. Interestingly, the very same mutations were still associated with an increased HCC risk, even after HCV elimination [32].

### 3.2. NS3

The NS3 protein of HCV is a multifunctional protein that acts as viral protease, RNA helicase and nucleoside triphosphatase (NTPase) during the viral life cycle [33]. Its serine protease activity cleaves the HCV polyprotein at four distinct sites to generate NS4A, NS4B, NS5A and NS5B [34]. The RNA helicase and NTPase function of NS3 are essential components of the HCV replication complex [35]. The direct role of NS3 as an inducer of cell transformation was initially demonstrated in vitro by the overexpression of NS3 in NIH 3T3 fibroblasts. The subsequent injection of these cells into nude mice led to the formation of ectopic tumors [36]. Moreover, NS3 promotes liver disease progression by stimulating hepatic fibrosis in HCV-infected chimeric mice, which can be attenuated by treatment with an anti-NS3 antibody [37].

### 3.3. NS5A

The viral phosphoprotein NS5A has a key role during RNA replication and virion assembly [38] and confers viral resistance to interferon (IFN) [39,40]. It is a driver of liver disease, inducing hepatic steatosis, as demonstrated in NS5A transgenic mice [41]. Transgenic mice expressing NS5A are protected against hepatic apoptosis following tumor necrosis factor alpha (TNF-α) injection [42]. However, NS5A expression alone is not sufficient to induce liver tumors [43,44], suggesting that its oncogenic role results from its interaction with additional cancer-related pathways [45] or in the context of co-morbidities. For example, NS5A transgenic mice develop liver cancer if fed with high-fat diet by exhibiting a pronounced mesenchymal phenotype [46]. NS5A hyperphosphorylation is essential for its function during the viral life cycle, indicating a tight interaction with host signaling pathways [18,33,47,48].

## 4. HCV-Induced Proliferative Signaling Associated with Liver Disease

**Figure 1:**
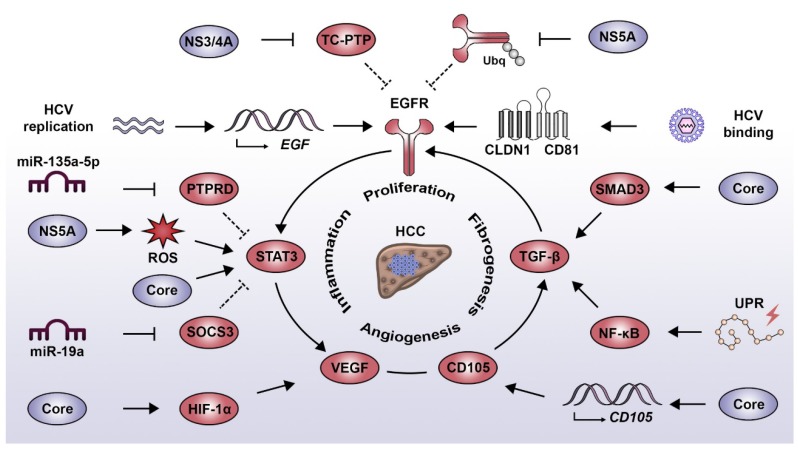
Interplay between HCV-induced oncogenic signaling pathways. EGF pathway: HCV binding to its entry receptor complex (i.e., CLDN1/CD81) induces EGFR phosphorylation. This is sustained by the action of NS3/4A and NS5A which negatively regulate the phosphatase TC-PTP and the process of EGFR degradation, respectively. Additionally, HCV replication has been linked to the increased expression of EGF and activation of the TGF-β pathway, both contributing to EGFR signaling. STAT3 pathway: STAT3 activation results from the direct action of the core protein and indirectly via EGFR activation and the NS5A protein, which favors the production of ROS. Moreover, HCV employs miR-135a-5p and miR-19a to decrease the expression of the negative STAT3 regulators PTPRD and SOCS3, respectively. TGF-β pathway: HCV induces the activation of the TGF-β pathway by intermediary of the UPR, which favors NF-κB activity and via the core protein, which directly interacts with SMAD3. The HCV-mediated expression of endoglin (CD105) favors activation of the TGF-β pathway and the induction of angiogenesis signaling. VEGF pathway: HCV core induces the activation of HIF-1α, which leads to an increased expression of VEGF. Similarly, an increased VEGF expression is promoted by HCV via the STAT3-dependent activation of androgen receptor. Abbreviations: CLDN1, claudin 1; EGF, epidermal growth factor; EGFR, epidermal growth factor receptor; HCC, hepatocellular carcinoma; HCV, hepatitis C virus; HIF-1α, hypoxia-inducible factor 1 alpha; NF-κB, nuclear factor kappa B; PTPRD, protein tyrosine phosphatase receptor type delta; ROS, reactive oxygen species; SMAD3, SMAD family member 3; SOCS3, suppressor of cytokine signaling 3; STAT3, signal transducer and activator of transcription 3; TC-PTP, T cell protein tyrosine phosphatase; TGF-β, transforming growth factor beta; UPR, unfolded protein response; VEGF, vascular endothelial growth factor.

HCV infection has been reported to induce a wide range of cell signaling alterations, which directly or indirectly contribute to the development and progression of liver diseases [49,50]. In the following paragraphs, we illustrate this aspect by describing the relationship between HCV and four major signaling drivers that play an important role in liver disease progression towards HCC: the epidermal growth factor (EGF), signal transducer and activator of transcription 3 (STAT3), transforming growth factor beta (TGF-β) and vascular endothelial growth factor (VEGF). In its evolution, HCV diverted these pathways to promote its replication and persistence with important consequences to viral pathogenesis and liver disease (Figure 1).

### 4.1. EGF Signaling Pathway

In the context of HCV infection, one of the best characterized signaling components associated with the development and progression of liver disease is the epidermal growth factor receptor (EGFR) pathway [51]. EGFR is an essential host factor regulating entry of the virus into hepatocytes [52] and is chronically deregulated in the liver of HCV-infected patients [52,53]. HCV has developed multiple mechanisms to induce and maintain EGFR signaling. In the early stages of the viral life cycle, HCV binding to its entry receptor complex, i.e., CD81 and claudin-1 (CLDN1), induces EGFR phosphorylation [54] and downstream signaling [55], thereby facilitating viral particle internalization. EGFR activity is prolonged by the NS5A-mediated alteration of EGFR trafficking [56] and by stimulated Netrin-1 expression, which impedes EGFR recycling [57]. Moreover, HCV replication itself promotes the expression of the receptor ligand EGF [58]. Additionally, NS3/4A activity induces the proteolytic cleavage of the EGFR phosphatase T cell protein tyrosine phosphatase (TC-PTP), thus sustaining EGFR activation [59]. Beyond viral entry and replication, persistent EGFR signaling contributes to the viral evasion from the antiviral activity of type I IFNs. EGFR activity suppresses the expression of suppressor of cytokine signaling 3 (SOCS3), a negative feedback regulator of STAT3 resulting in a blunting of the antiviral IFN response [60]. The persistent virus-induced signal transduction by the EGFR pathway leads to drastic changes not only in the infected hepatocytes but also in the immediate liver microenvironment with important consequences to liver pathogenesis. EGF expression is a main driver of liver fibrosis and HCC [61] and is part of a prognostic transcriptional signature associating with HCC development and patient survival [62,63,64]. Moreover, a recent study has shown that HCV infection induces the EGFR-dependent expression of invadopodia-related genes, therefore enhancing intra- and extrahepatic HCC dissemination in vivo [65].

### 4.2. STAT3 Signaling Pathways

Following liver injury, the release of inflammatory cytokines induces the activation of signaling pathways which prime hepatocytes for proliferation and allows liver regeneration via compensatory hyperplasia. Similarly to EGFR, STAT3 plays a central role in the tight regulation of this process, as observed in animal models of partial hepatectomy [66]. However, in the context of a persistent inflammatory response, as observed during HCV infection, this sustained STAT3 activation favors liver disease development [67]. STAT3 is also a host factor promoting HCV replication [55,68]. HCV induces STAT3 activation in a direct manner via its interaction with the core protein [69] and indirectly through NS5A and the production of ROS [70]. Moreover, miR-135a-5p, which is a host factor for HCV by itself [71], is upregulated following HCV infection targeting the STAT3 phosphatase protein tyrosine phosphatase receptor type delta (PTPRD) for degradation [72]. Consequently, impaired PTPRD expression leads to an enhanced STAT3 transcriptional activity [72]. Overactivated STAT3 has been shown to control microtubule dynamics through contact inhibition with stathmin, thus enhancing intracellular trafficking of the virus and increasing replication [68]. The activation of STAT3 is not limited to hepatocytes, as it has been demonstrated that HCV-infected cells secrete miR-19a in exosomes, which promotes STAT3 phosphorylation in hepatic stellate cells (HSCs) via downregulation of SOCS3. This stimulates HSC activation and virus-induced pro-fibrotic TGF-β1 signaling [73] and thus, contributes to liver disease progression and cirrhosis [74]. Consistently, STAT3 activation is enhanced in more aggressive HCC tumors [75].

### 4.3. TGF-β Signaling Pathway

The activation of the TGF-β pathway has been established as one of the main cellular signaling events associated with the development of liver fibrosis [76]. In HCCs, this cytokine has a dual role as tumor suppressor in the early stages of HCC development and as tumor promoter at later stages by stimulating the expression of antiapoptotic genes [77,78]. Interestingly, the dissection of the molecular mechanism attributed the oncogenic effect of TGF-β to its capacity to activate the EGFR pathway [79,80]. HCV infection stimulates TGF-β and EGFR signaling, which in turn promotes HCV entry and replication [52,81]. Consistently, the expression level of TGF-β1 decreases following viral clearance [82]. On the one hand, HCV indirectly favors TGF-β signaling via nuclear factor kappa B (NF-κB) by inducing an unfolded protein response (UPR), which is triggered by chronic infection and membrane remodeling [83]. On the other hand, HCV directly induces TGF-β signaling via the interaction of HCV core with SMAD family member 3 (SMAD3) [84] and via the core-stimulated expression of endoglin (CD105) on the surface of hepatocytes [85]. Endoglin is a component of the TGF-β receptor complex favoring signaling pathways related to liver fibrosis and tumor growth [85]. Moreover, endoglin also plays a role in signal transduction relevant to angiogenesis and is highly expressed in the vasculature of HCC tumors and endoglin expression is correlated with a poor prognosis [86].

### 4.4. VEGF Signaling Pathway

A key regulator of angiogenesis is VEGF signaling, which is deregulated in the majority of solid malignancies, since the growth of liver tumors requires the formation of new blood vessels to cope with the increased metabolic demands and with tissue hypoxia [87]. The involvement of this process during HCV infection is highlighted by the higher micro-vessel density in livers of HCV-infected patients as compared to chronic hepatitis B patients [88]. HCV induces the formation of new vasculature by multiple mechanisms such as the core-mediated activation of hypoxia-inducible factor 1 alpha (HIF-1α), which leads to the increased expression of VEGF [89]. Moreover, the HCV-induced activation of STAT3 enhances androgen receptor transcriptional activity, which also results in an increased expression of VEGF [90]. VEGF signaling has a proviral effect, facilitating HCV entry by altering occludin localization and by perturbing tight junction integrity [91]. This is potentially relevant to HCV-associated HCC, since the tissue and serum levels of VEGF correlate with patient survival after tumor resection [92].

## 5. Immune-Mediated Contribution to Liver Disease Progression during Chronic HCV Infection

**Figure 2:**
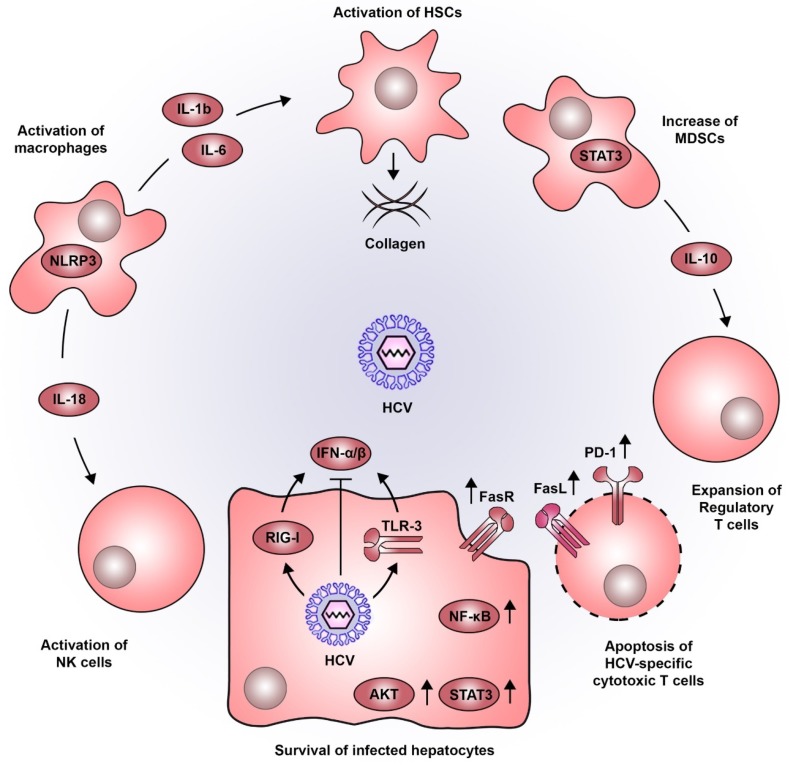
Pro-oncogenic inflammatory microenvironment induced by HCV. HCV infection in hepatocytes is detected by viral sensors such as RIG-I and TLR3, leading to the production of type I IFNs. As for most viruses, HCV has developed a wide variety of strategies to dampen this antiviral response. The persistent inflammatory environment in the liver combined with the action of viral proteins, establishes a sustained activation of signaling pathways associated to cell survival (e.g., STAT3, AKT, NF-κB and FasR). The sensing of HCV-infected hepatocytes by macrophages triggers NLRP3 inflammasomes, inducing the secretion of IL-18 which activates NK cells. Moreover, IL-1b and IL-6 produced by macrophages favor the activation of HSCs which are central components in the progressive deposition of collagen associated to liver cirrhosis. Additionally, STAT3 also plays a role in the development of MDSCs which produce IL-10 and favor the expansion of regulatory T cells. This altered immune response, is further accentuated by the increased expression of PD-1 and FasL, impairing cytotoxic T lymphocyte function and inducing their apoptosis. Abbreviations: AKT, AKT serine/threonine kinase; FasL, Fas ligand; FasR, Fas receptor; HCV, hepatitis C virus; HSCs, hepatic stellate cells; IFN, interferon; IL, interleukin; MDSCs, myeloid-derived suppressor cells; NF-κB, nuclear factor kappa B; NK, natural killer; NLRP3, NOD- LRR- and pyrin domain-containing protein 3; PD-1, programmed cell death 1; RIG-I, retinoic acid-inducible gene I; STAT3, signal transducer and activator of transcription 3; TLR3, toll like receptor 3.

HCV does not possess a latent phase in its life cycle and is considered to be largely noncytopathic, although also induction of apoptosis has been described [93]. It therefore poses a constant challenge to liver homeostasis, causing stress and inflammation. Triggered by innate immune responses, cytokine-stimulated non-parenchymal cells form a proinflammatory microenvironment as a major determinant of liver disease progression from fibrosis to cirrhosis and HCC (Figure 2). Indeed, 70–90% of all HCCs develop after a long history of liver disease due to chronic inflammation [94].

During viral infections, pathogen-associated molecular patterns (PAMPS) are recognized by innate immune sensors, i.e., toll-like receptors (TLRs), retinoic acid-inducible gene I (RIG-I), and cyclic GMP-AMP synthase (cGAS), triggering a rapid IFN type I response against infection [95]. As a prototype positive-stranded RNA virus, HCV is mainly recognized by the TLR3 and RIG-I, although it has developed several mechanisms to evade innate immune sensing and to blunt the resulting IFN response (reviewed in more detail in [96]). During chronic infection, HCV triggers TLR3 signaling also in monocytes and macrophages leading to the secretion of proinflammatory cytokines including interleukins (IL) and an activation of the inflammasome [97] without IFN induction. In vitro data suggest that sensing of HCV-infected hepatocytes by macrophages triggers NLR3P inflammasomes and induces IL-18 secretion, which activates natural killer (NK) cells [98]. Since the host fails to overcome HCV infection, the persistent deregulation of immune factors such as IFN signaling, activation of NF-κB, TNF-α and IL-6-mediated signaling were found to be significantly associated with a poor prognosis for HCC development [62] and thus, presumably contribute to liver disease progression.

In addition to the above-mentioned pro-viral and proliferative role of STAT3 signaling, this pathway also exhibits an important role during inflammation. The proinflammatory cytokines IL-6 and TNF-α activate the transcription factors STAT3 and NF-κB, which if persistently stimulated can aggravate liver disease progression and HCC development [99]. NF-κB signaling induces IFN-β production, which triggers JAK/STAT signaling in neighboring cells and consequently leads to the expression of antiviral IFN stimulated genes. However, these events are directly targeted by HCV proteins blunting the innate antiviral response. In contrast, HCV proteins core and NS5A also block apoptosis of infected cells by activation of AKT serine/threonine kinase (AKT) and NF-kB [100]. Chronic HCV infection stimulates the expression of Fas cell surface death receptor (FAS) in hepatocytes promoting cell survival of infected cells and adding to the pro-oncogenic environment caused by the virus. The same mechanism triggers the apoptosis of inflammatory T cells [101], and thus, promotes the survival of infected pro-oncogenic cells. Chronic HCV infection results in the dysregulation of the balance between Fas receptor (FasR, CD95) and ligand (FasL, CD95L). FasL positive T cells have been shown to interact with FasR-exposing hepatocytes, which results in liver cell apoptosis. During chronic HCV infection, upregulation of FasR expression on hepatocytes as well as FasL upregulation in T cells significantly correlate with the severity of liver inflammation [102,103]. In addition, FasR expression is almost undetectable in HCC [104], suggesting a reduced susceptibility towards T cell-mediated cytotoxicity, which potentially results in an increased survival of tumorigenic cells.

Contributing to this effect is also the lack of an effective T cell response during chronic infection. The constant and prolonged exposure of T cells to HCV antigens causes exhaustion, especially of CD8+ T cells [105,106]. Additionally, HCV-specific T cells undergo massive apoptosis during the chronic phase of infection, which in turn may contribute to the chronic inflammatory environment during HCV infection [107]. A key inhibitory marker on exhausted T cells is programmed cell death 1 (PD-1). Its expression on exhausted T cells is at least partially maintained even following DAA cure [108,109], potentially due to epigenetic modifications induced by long term exposure to HCV. Additionally, multiple reports suggest that immune cells, including T cells can also be productively infected by HCV [12,13,14,15], although it remains unclear to which degree this might affect the HCV-specific immune response [13,15,16]. HCV infection has been linked to the presence of myeloid-derived suppressor cells (MDSCs), a population of myeloid cells that negatively regulates the function of NK, CD4+ and CD8+ T cells [110]. Again, STAT3 activation is a central driver in the development of MDSCs, as it induces the expression of suppressive genes, i.e., IL-10, which in turn favor the expansion of regulatory T (Treg) cells [111,112]. Evidence suggests that MDSCs increase tumor burden and metastasis rate in liver cancer animal models and patients with HCC [113].

HSCs are important regulators for the liver extracellular matrix and wound healing. During chronic injury, activated stellate cells are the main drivers of liver fibrosis, which is characterized by an excessive deposition of collagen scar tissue. If unstopped, this process is a main cause of impaired liver function and cirrhosis [114]. Importantly, NF-κB signaling and the secretion of proinflammatory cytokines, i.e., IL-6 and IL-1b into the microenvironment of infected hepatocytes also activate HSCs [115,116,117,118] and thus cause an acceleration of collagen deposition into the extracellular space and liver fibrosis.

## 6. Virus-Induced Epigenetic Dysregulation

It became evident that HCV not only promotes pro-oncogenic events and liver fibrosis but also perturbs epigenetic regulatory circuits in hepatocytes with important long-term consequences to the host. Epigenetic homeostasis via DNA methylation of regulatory elements, post-translational modifications of histones, and non-coding RNA (ncRNA)-mediated gene silencing is essential for the memory of genetic regulation in the context of environmental conditions [119]. It is therefore widely accepted that epigenetic dysregulation is an important factor in the development of pathologies including cancer [120,121].

### 6.1. CpG Methylation of Host DNA

During chronic HCV infection, a combination of direct and indirect factors can influence the epigenome. Viral proteins, such as core and NS5A possess a nuclear localization signal and can be detected in the nucleus and thus being close to host DNA [122,123,124,125]. HCV core protein markedly increases the expression of DNA methyltransferase (DNMT)-1 and histone deacetylase (HDAC)-1 [126] and has been suggested to cause epigenetic silencing of tumor suppressor gene expression by DNA-methylation of cytosine-phospho-guanine (CpG) dinucleotides in regulatory gene elements [127]. Tumor suppressor genes silenced by core include secreted frizzled-related protein (SFRP) [126], which promotes epithelial-mesenchymal transition (EMT) and deregulates Wnt/ß-catenin signaling as major pathways involved in HCC development [128]. HCV-induced hypermethylation of cancer-related genes such as *APC*, *p73*, *p14*, and *O^6^MGMT* (summarized in [129]) and perturbed methylation in repetitive DNA elements have been observed [130], suggesting a relevance for HCV-related HCC.

### 6.2. Non-Coding RNA

Gene expression is also regulated by ncRNAs [119], known to affect biological processes encompassing differentiation, proliferation, cell death, and cancer [131]. ncRNAs comprise small ncRNA (sncRNA) and long ncRNA (lncRNA), which are both involved in HCC, as reviewed in [131]. Among tens of thousands of lncRNAs many are reportedly related to HCC, including HOTAIR [132,133] and PVT1 [134] functioning via epigenetic repression and cell cycle progression. sncRNAs, in particular miRNAs are relevant in HCCs, which besides small nucleolar RNAs (snoRNAs) and piwi-interacting RNAs (piRNAs) have been studied most extensively [119]. miRNAs are of 18-25 nucleotides length and regulate gene expression on the post-transcriptional level. They bind to complementary sequences in the 3′-untranslated region of mRNAs, which results in a translational suppression or degradation [135]. HCV affects miRNA expression with important impact on liver pathogenesis [71,72,136]. In addition to the above-mentioned regulatory roles of miR-19a and miR-135-5p in virus-induced signaling, miR-146a-5p [136] and the liver specific miR-122 [17] are also deregulated by HCV infection and are linked to liver disease and HCC development [131].

### 6.3. Histone Modifications

Posttranslational modifications of histones regulate the binding affinity of DNA to histone proteins and therefore influence accessibility of genes within chromatin to the transcriptional machinery. These modifications comprise acetylation, methylation, phosphorylation, and ubiquitination [127]. A recent combined genome-wide analysis of the HCV-related epigenome using Chip-Seq and transcriptomic data (RNA-Seq) from HCV-infected liver tissues highlighted a potential role of virus-induced acetylation of histone H3 at position lysine 27 (H3K27Ac) in liver pathogenesis [137,138]. H3K27Ac is considered to be an activation mark promoting the transcription of associated genes by distinguishing active enhancers from inactive/poised enhancer elements [139]. Strikingly, these studies showed that chronic HCV infection induces specific genome-wide changes in H3K27Ac, which correlated with the expression of known cancer risk genes [137]. Similar observations have been made in HCV-infected cell culture for histone H3 lysine 9 acetylation (H3K9Ac) activation mark [140]. Importantly, since epigenetic regulation of gene expression can be considered as a lasting “epigenetic memory” [141], it may suggest that HCV-induced changes persist even after viral cure. Indeed, an epigenetic viral footprint can be detected in HCV-cured cell culture, chimeric mice and patients, which are correlated with elevated cancer-risk gene expression and lower patient survival after surgical resection of HCV-associated HCC [137,140]. This footprint is certainly a combination of direct, virus-induced effects and indirect fibrosis-related effects, which in patients with chronic HCV infection are closely linked. The comparison of H3K27Ac Chip-Seq and RNA-Seq profiles in HCV-cured fibrotic patient livers with livers of non-fibrotic HCV-cured chimeric mice yielded an HCV-specific persistent epigenetic and transcriptomic “footprint” of 65 cancer-risk genes [137]. Moreover, a fibrosis-associated “footprint” of 1693 cancer-risk genes was identified in fibrotic livers of HCV patients and in patients with non-alcoholic fatty liver disease (NAFLD). Dysregulation of a subset of 25 genes of this fibrotic epigenetic footprint is termed the prognostic epigenetic signature (PES) and is predictive for cancer risk in patients [138]. Importantly, epidrugs can remove these epigenetic persistent footprints and reduce cancer risk in cell culture and animal models [138], highlighting a potential for future chemopreventive strategies [49].

## 7. Clinical Implications for Biomarker Discovery to Predict HCV-Induced HCC Risk

Only 30 years after its discovery, HCV is now a curable disease thanks to an unprecedented effort of the combined work of scientists, physicians and pharma. However, the treatment-induced viral cure cannot fully eradicate HCV-associated complications and HCC risk especially in patients with advanced liver disease [142]. Due to the relatively long delay between virus-induced liver injury and the development of HCC, it is assumed that the epidemiologic peak of HCV-associated liver disease and HCC is yet to come. This highlights two urgent unmet medical needs for the clinical management of patients with SVR: reliable biomarkers to identify the fraction of SVR patients with elevated HCC risk and efficient and safe chemopreventive strategies targeting virus-specific pro-oncogenic pathways, epigenetic footprints and liver fibrosis to help these patients.

Long-term chronic infection with HCV causes liver disease progression from fibrosis to liver cirrhosis and the occurrence of pre-neoplastic lesions with a certain accumulation of genetic mutations including telomerase promoter, p53 and beta catenin pathway [143,144]. This relative diversity of individual defects makes it difficult to identify a common and reliable biomarker predictive for HCC risk. A 186-gene transcriptional signature has been identified in non-tumor tissue adjacent to HCC lesions and in HCV-related early-stage cirrhosis, which is predictive for HCC risk [62,63]. This so-called prognostic liver signature (PLS) can recapitulate HCC risk in patients independent from the underlying liver disease etiology [64]. Recently discovered signatures based on virus-induced epigenetic modifications [137], such as the PES [138], provide a novel perspective to assess residual HCC risk in HCV patients after SVR and allow to select these patients for clinical trials to evaluate chemoprevention of HCC.

Many of the above-mentioned virus-induced pro-oncogenic pathways are also drivers in non-HCV-associated liver disease. Namely the pro-inflammatory, proliferative and pro-fibrinogenic signaling pathways EGF, IL-6, and TGF-ß are also part of the etiology independent biomarker signature PLS [64]. Chronic HCV infection has been associated with a range of extrahepatic complications, such as mixed cryoglobulinemia and B cell lymphomas [145]. Although the mechanisms involved are not fully understood, it has been suggested that TGF-β and IL-6 play a potential role in the development of these complications [146,147]. It is thus conceivable that persistent HCV-induced signaling alterations and deranged cytokine production may promote the development of extrahepatic manifestations in immune cells [148]. Several compounds targeting these signaling pathways have been suggested for chemoprevention of HCC (reviewed in detail in [149,150]) and targeting HCV-relevant pathways to treat established HCC have been suggested [151,152,153]. Therefore, learning from HCV-induced liver disease will help to develop novel diagnostics and personalized therapeutic concepts that may be useful to prevent and potentially also to treat HCC from other related liver disease etiologies like NAFLD.
